# Essentials of Human Physiology

**Published:** 1909-09

**Authors:** 


### Reviews of Books.

**Essentials of Human Physiology.** By D. NOËL PATON, M.D. Pp. xv, 477. Third Edition. Edinburgh and London: William Green & Sons. 1907.

**Physiological Principles in Treatment.** By W. LANGDON BROWN, M.D. Pp. vii, 344. London: Baillière, Tindall & Cox. 1908.

The publication of a new edition of Dr. Noël Paton's excellent book and the appearance of a small volume by Dr. Langdon Brown on the treatment of certain classes of disease, bring into a prominence—which is not more than they deserve—the services rendered to practical therapy by the pure science of physiology.

The earlier editions of Dr. Noël Paton's book have already been reviewed in this *Journal*, and his name is well known to all students of physiology, so that it is hardly necessary to deal with his work in detail. We may note, however, that several portions have been newly written for this edition, for instance, the description of the proteins, which is brought into line with Fischer's recent researches, and with the nomenclature adopted by the Joint Committee of the Chemical and Physiological Societies. Much of the neurological work has also required considerable revision. To those who like succinctness of expression, Dr. Paton's "Essentials" is altogether a book to be commended; but they must remember that the "Essentials" are to be regarded as relative to the study of medicine, and not to that of physiology as a science in itself.

Senior students, and still more medical practitioners, should all read Dr. Langdon Brown's book. It is clearly written; no unnecessary padding has been introduced; the style is pleasantly emphatic, and the practical deductions plainly and conclusively stated. The author is to be congratulated on having carried out his idea so well and the general practitioner on having acquired a volume of so useful and stimulating a character.

Taken together, these books point an admirable moral as to the teaching of physiology to the medical student. In the first place, as physiology is a growing science it cannot be taught by excerpts, but must be taken as a whole. Detail may often be curtailed, and in especial the pros and cons of past controversies may be omitted altogether; but all sections must be adequately represented in the student's mind if he is, as a practitioner, to be in a position to avail himself of such applications of physiology to medicine which may from time to time appear.

Dr. Langdon Brown's book shows how very various are the lines along which the practical application of physiology has developed, and it is not going too far to say that, should a choice be necessary, it would be better for the student to omit the anatomy of one of the limbs from his curriculum than to omit one of the main branches of physiological knowledge, however far it may now seem to be removed from the everyday practice of medicine.

